# Dataset of leaf inclination angles for 71 different *Eucalyptus* species

**DOI:** 10.1016/j.dib.2020.106391

**Published:** 2020-10-09

**Authors:** Jan Pisek, Kairi Adamson

**Affiliations:** Tartu Observatory, University of Tartu, Observatooriumi 1, Tõravere, 61602 Tartumaa, Estonia

**Keywords:** Leaf inclination angle distribution, Optical canopy instrumentation, Leveled digital photography

## Abstract

The leaf inclination angle distribution is an important parameter in models useful for understanding forest canopy processes of photosynthesis, evapotranspiration, radiation transmission, and spectral reflectance. Yet, despite the strong sensitivity of many of these models to variability in leaf inclination angle distribution, relatively few measurements have been reported for different tree species in literature and databases such as TRY, and various assumptions about leaf inclination angle distribution are often made by modellers.

Here we provide a dataset of leaf inclination angles for 71 different Australia-native *Eucalyptus* species measured in 13 botanical gardens around the world. Leaf inclination angles were measured using a leveled digital camera approach. The leaf angle measurements were used to estimate corresponding Beta distribution parameters and to assign the appropriate classic type of leaf inclination angle distribution. The data can be used to parameterize leaf angle distributions in e.g., physically-based reflectance models, land surface models, and regional carbon cycle models.

## Specifications Table

**Table:** 

Subject	Agricultural and Biological Sciences/Plant Science
Specific subject area	Anatomy, ecophysiology of *Eucalyptus* plant species and radiative transfer models
Type of data	Table
How data were acquired	Leaf angles were obtained via analysis of images taken with leveled digital photography. Cameras used: Nikon CoolPix 4500 digital camera (4MP), leveled, tripod-mounted; Sony Xperia Z5 Compact phone equipped with 23MP 1/2.3-inch multi-aspect BSI CMOS sensor, paired with an F2.0 lens, hand-balanced. Image processing software: ImageJ (http://imagej.nih.gov/ij/).
Data format	Raw Analysed R code
Parameters for data collection	Leaf angles were estimated for leaves with their surfaces oriented approximately perpendicular to the viewing direction of the digital camera.
Description of data collection	Series of leveled digital images of the tree crowns were taken during calm conditions to prevent wind effects on leaves along a vertical tree profile.
Data source location	Australian National Botanic Gardens, Canberra, ACT, Australia Blue Mountains Botanic Garden, Mount Tomah NSW, Australia National Arboretum Canberra, Canberra. ACT, Australia Royal Botanic Gardens Victoria - Melbourne Gardens, Melbourne, VIC, Australia Royal Botanic Gardens, Sydney, NSW, Australia Royal Tasmanian Botanical Gardens, Hobart, TAS, Australia The Australian Botanic Garden, Mount Annan, NSW, Australia The Jerusalem Botanical Gardens, Jerusalem, Israel Jardí Botànic de València, València, Spain Royal Botanic Gardens, Kew, the United Kingdom San Francisco Botanical Garden, San Francisco, CA, USA The Huntington Botanical Gardens, Pasadena, CA, USA University of California Botanical Garden at Berkeley, Berkeley, CA, USA
Data accessibility	Repository name: Mendeley data Data identification number: 10.17632/h76nbndxt6.4 Direct URL to data: https://data.mendeley.com/datasets/h76nbndxt6/4

## Value of the Data

• Leaf inclination angle distribution is an important parameter which influences spectral reflectance and radiation transmission properties of vegetation canopies, and hence interception, absorption and photosynthesis. To date, relatively few measurements of leaf inclination angle have been reported for different tree species, *Eucalyptus* species in particular.
• The data can be used to parameterize leaf inclination angle distributions in e.g., physically-based reflectance models, land surface models, and regional carbon cycle models.
• The data can be used as a plant functional trait and in functional diversity analyses.
• The data can provide information for understanding light use efficiency and photosynthetic strategies of different plant species.
• The data can be used to compare measurements performed for the same species by other studies and/or other methods.

## 1 Data Description

This article reports a dataset of leaf angle measurements for 71 different, Australia-native *Eucalyptus* species collected in 13 botanical gardens (Table 1). Leaf inclination angles were measured using a leveled digital camera approach [1]. Images were taken during calm conditions to prevent wind effects on leaves [2]. Depending on the location the images were taken either with a Nikon CoolPix 4500 digital camera (4MP) or a Sony Xperia Z5 Compact phone equipped with 23MP 1/2.3-inch multi-aspect BSI CMOS sensor paired with an F2.0 lens. Leaves were measured in all the azimuth directions as conditions permitted, and along the vertical profile. The data consist of one raw data file (“Pisek_Adamson_2020_DiB.csv”) with 6646 lines (one header line; individual leaf angle measurements) and 10 columns (variables). The data format corresponds to the one used for reporting leaf angle measurements in TRY plant trait database [3]. The column names and definitions of variables are provided in Table 2. The resulting statistical characteristics of leaf inclination angle distributions for each studied species are provided in Table 3 as well as the file “Pisek_Adamson_2020_DiB_processed.csv” in the supplementary material. The statistical characteristics of leaf inclination angle distributions for each studied species were obtained with a R code (“getLIAD.R”), sourced from the original code by [4]. The example input file format (“input_example_LIA.csv”) is also provided.

**Table 1:** Locations of botanical gardens where the measurements were taken. Lat - Latitude, Lon - Longitude, Date of measurements provided in YYYYMMDD format.

Botanical garden	Lat	Lon	Date of measurements
Australian National Botanic Gardens, Canberra, ACT, Australia	−35.276	149.108	20,190,222
Blue Mountains Botanic Garden, Mount Tomah NSW, Australia	−33.539	150.421	20,190,217
National Arboretum Canberra, Canberra. ACT, Australia	−35.287	149.069	20,190,222
Royal Botanic Gardens Victoria - Melbourne Gardens, Melbourne, VIC, Australia	−37.829	144.978	20,130,719
Royal Botanic Gardens, Sydney, NSW, Australia	−33.864	151.217	20,190,213
Royal Tasmanian Botanical Gardens, Hobart, TAS, Australia	−42.865	147.330	20,130,728
The Australian Botanic Garden, Mount Annan, NSW, Australia	−34.071	150.766	20,190,224
The Jerusalem Botanical Gardens, Jerusalem, Israel	−31.770	35.200	20,150,227
Jardí Botànic de València, València, Spain	−39.477	−0.386	20,171,107
Royal Botanic Gardens, Kew, the United Kingdom	51.478	−0.295	20,171,019
San Francisco Botanical Garden, San Francisco, CA, USA	37.767	−122.470	20,151,214
The Huntington Botanical Gardens, Pasadena, CA, USA	34.128	−118.116	20,121,209
University of California Botanical Garden at Berkeley, Berkeley, CA, USA	37.874	−122.238	20,131,210

**Table 2:** Data description including column names and variable definitions in “Pisek_Adamson_2020_DiB.csv”.

Column name	Description
Species	Latin name for the species
Latitude	Location latitude (in decimal degrees)
Longitude	Location longitude (in decimal degrees)
Altitude (m a.s.l.)	Location altitude (in meters above sea level)
Sampling Date	(mm/dd/yy)
Exposition	botanical garden/alley/natural forest
Maturity	mature/seedling
Plant Growth Form	mallee, tree, small tree, shrub
Comments, Methods	angles measured at whole tree level using leveled digital photo method; leaf angles reported as differences from a horizontal surface (i.e. flat horizontal leaf = 0°, vertically oriented leaf = 90°).
Reference	Corresponding publication
Measurement	in degrees, values 0–90

**Table 3:** Statistical characteristics (i.e., mean, standard deviation) of leaf angle distributions with two parameters μ, ν and classic type of leaf angle distribution of fitted Beta-distributions. PG – plagiophile, U – uniform, S – spherical, Er – erectophile. Table available as “Pisek_Adamson_2020_DiB_processed.csv” in the supplementary material.

Species name	Measurement location	Count	Mean	S.D.	u	v	Type
*Eucalyptus albopurpurea*	Jerusalem, IL	84	59.89	22.11	0.90	1.79	S
*Eucalyptus amplifolia*	NBG Canberra,, ACT, AU	83	76.65	13.31	0.71	4.07	Er
*Eucalyptus archeri*	Kew, GB	90	50.78	24.60	1.00	1.29	U
*Eucalyptus baeuerlenii*	NBG Canberra,, ACT, AU	83	69.23	21.97	0.46	1.52	Er
*Eucalyptus balladoniensis*	Pasadena, CA, USA	83	49.68	22.57	1.31	1.62	U
*Eucalyptus benthamii*	Canberra, ACT, AU	81	73.86	13.93	0.92	4.22	Er
*Eucalyptus caesia*	Melbourne, VIC, AU	100	66.58	16.67	1.20	3.41	Er
*Eucalyptus calycogona*	Pasadena, CA, USA	100	40.70	24.49	1.29	1.06	U
*Eucalyptus camaldulensis*	Hobart, TAS, AU	50	69.23	18.49	0.74	2.47	Er
*Eucalyptus camaldulensis* var. *Acuminata*	Jerusalem, IL	85	72.59	12.73	1.31	5.48	Er
*Eucalyptus chapmaniana*	Kew, GB	86	83.18	5.16	1.54	18.76	Er
*Eucalyptus coccifera*	Kew, GB	85	70.47	19.96	0.53	1.92	Er
*Eucalyptus coolabah*	Canberra, ACT, AU	87	55.01	22.71	1.06	1.67	S
*Eucalyptus copulans*	Mt. Annan, NSW, AU	80	51.74	25.81	0.84	1.13	U
*Eucalyptus crebra*	Mt. Annan, NSW, AU	83	67.80	17.95	0.91	2.77	Er
*Eucalyptus dalrympleana*	Kew, GB	85	79.17	10.83	0.76	5.55	Er
*Eucalyptus decurva*	Berkeley, CA, USA	66	60.04	19.80	1.20	2.40	S
*Eucalyptus delegatensis*	Kew, GB	78	80.87	6.31	1.78	15.76	Er
*Eucalyptus deuaensis*	Sydney, CA, USA	85	48.32	27.69	0.75	0.87	U
*Eucalyptus eremicola*	Jerusalem, IL	79	59.38	20.52	1.13	2.19	S
*Eucalyptus erythrocorys*	Valencia, ES	80	70.84	18.94	0.59	2.19	Er
*Eucalyptus erythronema*	Pasadena, CA, USA	90	43.97	24.37	1.23	1.18	U
*Eucalyptus eximia*	Pasadena, CA, USA	88	75.62	13.74	0.76	4.00	Er
*Eucalyptus ficifolia*	Pasadena, CA, USA	97	57.36	19.33	1.46	2.56	S
*Eucalyptus forrestiana*	Berkeley	85	62.36	19.10	1.14	2.58	Er
*Eucalyptus glaucescens*	Kew, GB	83	70.22	18.43	0.68	2.41	Er
*Eucalyptus gregsoniana*	Kew, GB	79	56.85	23.70	0.87	1.49	S
*Eucalyptus grossa*	Pasadena, CA, USA	82	43.77	27.58	0.85	0.81	U
*Eucalyptus guilfoylei*	Pasadena, CA, USA	97	46.08	19.22	2.19	2.29	PG
*Eucalyptus gunnii*	Kew, GB	82	61.71	24.54	0.60	1.30	S
*Eucalyptus haemastoma*	Mt. Annan, NSW, AU	85	67.29	16.51	1.16	3.44	Er
*Eucalyptus incrassata*	Jerusalem, IL	88	37.95	24.28	1.36	0.99	U
*Eucalyptus intertexta*	Canberra, ACT, AU	89	71.52	17.12	0.72	2.79	Er
*Eucalyptus jacksonii*	San Francisco, CA, USA	87	51.69	21.37	1.42	1.92	S
*Eucalyptus kruseana*	Pasadena, CA, USA	75	57.34	22.53	0.98	1.71	S
*Eucalyptus lacrimans*	Canberra, ACT, AU	100	57.68	19.76	1.36	2.42	S
*Eucalyptus lacrimans*	Pasadena, CA, USA	75	67.78	16.64	1.10	3.34	Er
*Eucalyptus laevopinea*	Mt. Annan, NSW, AU	82	65.64	24.86	0.43	1.16	S
*Eucalyptus langleyi*	Canberra, ACT, AU	85	63.80	21.23	0.79	1.92	Er
*Eucalyptus lansdowneana* ssp. *Albopurpurea*	Pasadena, CA, USA	83	38.36	24.68	1.29	0.96	U
*Eucalyptus leucoxylon*	Jerusalem, IL	93	53.78	25.84	0.77	1.15	S
*Eucalyptus litorea*	Jerusalem, IL	91	43.00	25.78	1.07	0.98	U
*Eucalyptus macrandra*	Pasadena, CA, USA	96	41.85	24.94	1.20	1.04	U
*Eucalyptus macrocarpa*	Mt. Annan, NSW, AU	12	60.60	14.52	2.43	5.01	Er
*Eucalyptus mannifera*	Canberra, ACT, AU	84	66.25	18.48	0.95	2.66	Er
*Eucalyptus michaeliana*	Canberra, ACT, AU	81	76.59	10.45	1.25	7.16	Er
*Eucalyptus microtheca*	Pasadena, CA, USA	84	77.79	10.08	1.13	7.22	Er
*Eucalyptus nitida*	Kew, GB	83	72.93	19.02	0.46	1.98	Er
*Eucalyptus morrisbyi*	Canberra, ACT, AU	78	58.85	24.54	0.71	1.34	S
*Eucalyptus neglecta*	Kew, GB	80	72.09	20.45	0.42	1.67	Er
*Eucalyptus nicholii*	Canberra, ACT, AU	88	69.57	19.72	0.60	2.05	Er
*Eucalyptus nicholii*	San Francisco, CA, USA	89	72.99	19.19	0.45	1.92	Er
*Eucalyptus oleosa*	Pasadena, CA, USA	84	46.45	25.63	1.01	1.07	U
*Eucalyptus oleosa*	Jerusalem, IL	100	48.19	25.18	1.01	1.17	U
*Eucalyptus parramattensis*	Mt. Annan, NSW, AU	80	74.87	17.13	0.48	2.38	Er
*Eucalyptus parvula*	Kew, GB	100	70.04	16.36	0.94	3.29	Er
*Eucalyptus parvula*	Canberra, ACT, AU	100	52.15	22.53	1.21	1.67	S
*Eucalyptus perriniana*	Kew, GB	78	79.75	12.38	0.49	3.84	Er
*Eucalyptus petiolaris*	Jerusalem, IL	56	38.54	22.82	1.61	1.20	U
*Eucalyptus pleurocarpa*	Mt. Annan, NSW, AU	76	68.87	21.47	0.51	1.65	Er
*Eucalyptus porosa*	Jerusalem, IL	91	68.18	19.07	0.75	2.34	Er
*Eucalyptus pulchella*	Kew, GB	100	67.40	17.68	0.97	2.90	Er
*Eucalyptus pulchella*	Melbourne, VIC, AU	91	49.93	21.23	1.53	1.91	S
*Eucalyptus pulverulenta*	Melbourne, VIC, AU	58	76.94	10.82	1.10	6.49	Er
*Eucalyptus raveretiana*	Mt. Annan, NSW, AU	78	71.18	17.35	0.72	2.73	Er
*Eucalyptus robusta*	Pasadena, CA, USA	98	49.83	18.64	2.13	2.64	PG
*Eucalyptus rodwayi*	Kew, GB	43	63.61	21.89	0.73	1.77	Er
*Eucalyptus scoparia*	Mt. Annan, NSW, AU	77	72.00	19.71	0.47	1.87	Er
*Eucalyptus scoparia*	Canberra, ACT, AU	77	71.13	16.10	0.88	3.30	Er
*Eucalyptus scoparia*	Canberra, ACT, AU	79	78.35	10.81	0.88	5.93	Er
*Eucalyptus shirleyi*	Pasadena, CA, USA	100	48.66	21.12	1.61	1.90	U
*Eucalyptus sideroxylon*	Hobart, TAS, AU	68	64.00	18.89	1.06	2.60	Er
*Eucalyptus stellulata*	Mt. Tobah, NSW, AU	80	77.52	12.83	0.68	4.20	Er
*Eucalyptus stoatei*	Pasadena, CA, USA	86	42.43	25.24	1.15	1.02	U
*Eucalyptus stricta*	Canberra, ACT, AU	86	40.42	24.04	1.36	1.11	U
*Eucalyptus subcrenulata*	Kew, GB	80	56.85	25.14	0.73	1.25	S
*Eucalyptus tereticornis*	Mt. Annan, NSW, AU	120	70.62	17.29	0.77	2.81	Er
*Eucalyptus tricarpa*	Canberra, ACT, AU	80	64.09	22.36	0.67	1.65	Er
*Eucalyptus urnigera*	Kew, GB	80	64.85	22.35	0.63	1.63	Er
*Eucalyptus viridis*	Jerusalem, IL	75	42.19	25.85	1.07	0.95	U

## 2 Experimental Design, Materials and Methods

### 2.1 Leaf inclination measurements and data processing

**Fig. 1:**
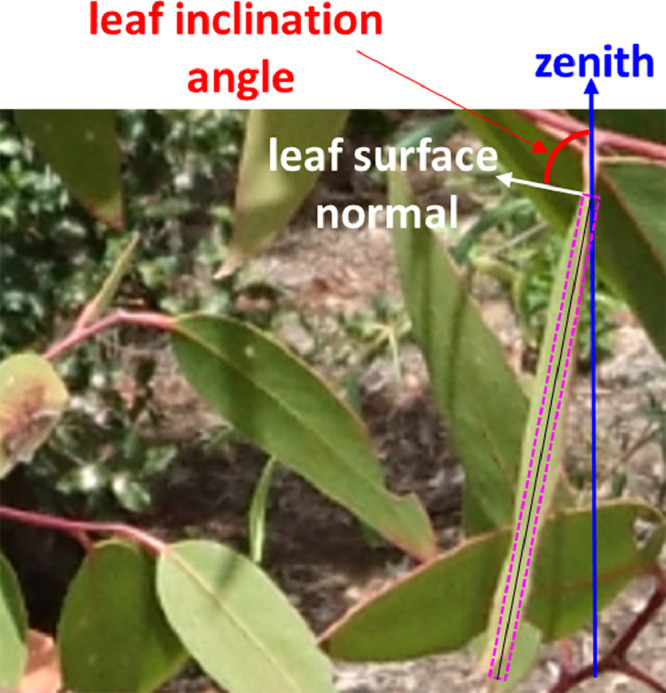
A schematic diagram of the protocol used to measure leaf inclination angle from leveled digital photography. The leaf plane is indicated by the line in a purple box.

The method proposed by [1] consists of acquiring leveled images of the canopy with a digital camera. A minimum of 75 leaf inclination angle measurements shall permit a statistically representative sample to characterize the leaf inclination angle distribution [5]. It shall be noted that the method is suited to broadleaf plant species [6]. The identification of the leaf plane, from which the leaf normal is measured, is required for the measurement of leaf inclination angle (Fig. 1). For this reason, the leaves oriented approximately perpendicular to the viewing direction of the camera (i.e., the leaves shown as a line in the image; Fig. 1) were selected for measurement of leaf angles. The leaf angles were measured using the ‘angle measurement tool’ of the freeware program ‘ImageJ’ (http://rsbweb.nih.gov/ij/). Although some level of uncertainty might be still present in individual leaf measurements due to user's subjectivity, the method was found quite robust in providing the same distributions of De Wit [7] irrespective of the user and their previous experience with measuring leaf inclination angles [8].

### 2.2 Estimation and assignment of beta distribution type

The measured leaf inclination angles were used to estimate the leaf inclination angle distribution for each species. A two-parameter Beta distribution [9] was previously identified as the most appropriate distribution to represent the probability density of *θ_L_* [10]:

(1) $$f\left(t\right)=\frac{1}{B\left(\mu ,\nu \right)}{\left(1-t\right)}^{\mu -1}t^{\nu -1}$$

where *t* = 2 *θ_L_/π* and θ*_L_* is expressed in radians. The Beta distribution *B(μ,ν)* is defined as:

(2) $$B\left(\mu ,\nu \right)=\int_{0}^{1}{\left(1-x\right)}^{\mu -1}x^{\nu -1}dx=\frac{\Gamma \left(\mu \right)\Gamma \left(\nu \right)}{\Gamma \left(\mu +\nu \right)}$$

where Γ is the Gamma function and *μ* and *ν* are two parameters of the Beta distribution, which are calculated as:

(3) $$\mu =\left(1-\bar{t}\right)\left(\frac{\sigma_{0}^{2}}{\sigma_{t}^{2}}-1\right)$$

(4) $$\nu =\bar{t}\left(\frac{\sigma_{0}^{2}}{\sigma_{t}^{2}}-1\right)$$

where $\sigma_{0}^{2}$ is the maximum standard deviation with an expected mean $\bar{t}$; $\sigma_{t}^{2}$ is the variance of *t* [10].

Leaf inclination angle distributions can be described with six common functions [7]: planophile, plagiophile, uniform, spherical, erectophile and extremophile. Horizontally oriented leaves are dominant in planophile canopies; plagiophile canopies are dominated by inclined leaves; uniform canopies possess about an equal proportion of leaf inclination angles for any angle; in spherical canopies, the relative frequency of leaf inclination angle is the same as for a sphere; erectophile canopies are dominated by vertically oriented leaves; extremophile distribution is a rather theoretical case, which would be characterized by both horizontally and vertically oriented leaves. All measured leaf inclination angle distributions were additionally classified by assigning them to the closest classical distribution type, since the classical distributions are widely used and easier to interpret than the Beta distribution parameters. Deviation of each leaf inclination angle distribution from the distributions suggested by de Wit *f*_*de* *Wit*_(*θ_L_*) was quantified with a modified version of the inclination index provided by [11]:

(5) $$\chi_{L}=\int_{0}^{\pi /2}\left|f\left(\theta_{L}\right)-f_{de\;Wit}\left(\theta_{L}\right)\right|d\theta_{L}$$

RStudio Version 1.0.153 has been used for all the data processing described above.

## Declaration of Competing Interest

The authors declare that they have no known competing financial interests or personal relationships which have or could be perceived to have influenced the work reported in this article.

## Appendix Supplementary materials

Image, application 1

Image, application 2

Image, application 3

Image, application 4
