# Supplementary material for: Dataset of leaf inclination angles for 71 different Eucalyptus species
Source: Data Brief. 2020 Oct 9;33:106391. doi: 10.1016/j.dib.2020.106391 (PMC7569295; doi:10.1016/j.dib.2020.106391)
Supplement: Supplementary file 2 [file mmc2.docx]

**Article Title: Dataset of leaf inclination angles for 71 different *Eucalyptus* species**

**Authors**

Jan Pisek^1^, Kairi Adamson^1^

**Affiliations**

1. Tartu Observatory, University of Tartu, Observatooriumi 1, Tõravere, 61602, Tartumaa, Estonia

**Corresponding author(s)**

Jan Pisek (janpisek@gmail.com)

**Abstract**

The leaf inclination angle distribution is an important parameter in models useful for understanding forest canopy processes of photosynthesis, evapotranspiration, radiation transmission, and spectral reflectance. Yet, despite the strong sensitivity of many of these models to variability in leaf inclination angle distribution, relatively few measurements have been reported for different tree species in literature and databases such as TRY, and various assumptions about leaf inclination angle distribution are often made by modellers.

Here we provide a dataset of leaf inclination angles for 71 different Australia-native *Eucalyptus* species measured in 13 botanical gardens around the world. Leaf inclination angles were measured using a leveled digital camera approach. The leaf angle measurements were used to estimate corresponding Beta distribution parameters and to assign the appropriate classic type of leaf inclination angle distribution. The data can be used to parameterize leaf angle distributions in e.g., physically-based reflectance models, land surface models, and regional carbon cycle models.

**Keywords**

Leaf inclination angle distribution, Optical canopy instrumentation, Leveled digital photography

**Specifications Table**

| **Subject** | Agricultural and Biological Sciences/Plant Science |
| --- | --- |
| **Specific subject area** | Anatomy, ecophysiology of *Eucalyptus* plant species and radiative transfer models |
| **Type of data** | Table |
| **How data were acquired** | Leaf angles were obtained via analysis of images taken with leveled digital photography.  Cameras used: Nikon CoolPix 4500 digital camera (4MP), leveled, tripod-mounted; Sony Xperia Z5 Compact phone equipped with 23MP 1/2.3-inch multi-aspect BSI CMOS sensor, paired with an F2.0 lens, hand-balanced.  Image processing software: ImageJ (http://imagej.nih.gov/ij/). |
| **Data format** | Raw  Analysed  R code |
| **Parameters for data collection** | Leaf angles were estimated for leaves with their surfaces oriented approximately perpendicular to the viewing direction of the digital camera. |
| **Description of data collection** | Series of leveled digital images of the tree crowns were taken during calm conditions to prevent wind effects on leaves along a vertical tree profile. |
| **Data source location** | Australian National Botanic Gardens, Canberra, ACT, Australia  Blue Mountains Botanic Garden, Mount Tomah NSW, Australia  National Arboretum Canberra, Canberra. ACT, Australia  Royal Botanic Gardens Victoria - Melbourne Gardens, Melbourne, VIC, Australia  Royal Botanic Gardens, Sydney, NSW, Australia  Royal Tasmanian Botanical Gardens, Hobart, TAS, Australia  The Australian Botanic Garden, Mount Annan, NSW, Australia  The Jerusalem Botanical Gardens, Jerusalem, Israel  Jardí Botànic de València, València, Spain  Royal Botanic Gardens, Kew, the United Kingdom  San Francisco Botanical Garden, San Francisco, CA, USA  The Huntington Botanical Gardens, Pasadena, CA, USA  University of California Botanical Garden at Berkeley, Berkeley, CA, USA |
| **Data accessibility** | Repository name: Mendeley data  Data identification number: 10.17632/h76nbndxt6.4  Direct URL to data: https://data.mendeley.com/datasets/h76nbndxt6/4 |

**Value of the Data**

- Leaf inclination angle distribution is an important parameter which influences spectral reflectance and radiation transmission properties of vegetation canopies, and hence interception, absorption and photosynthesis. To date, relatively few measurements of leaf inclination angle have been reported for different tree species, *Eucalyptus* species in particular.
- The data can be used to parameterize leaf inclination angle distributions in e.g., physically-based reflectance models, land surface models, and regional carbon cycle models.
- The data can be used as a plant functional trait and in functional diversity analyses.
- The data can provide information for understanding light use efficiency and photosynthetic strategies of different plant species.
- The data can be used to compare measurements performed for the same species by other studies and/or other methods.

**Data Description**

This article reports a dataset of leaf angle measurements for 71 different, Australia-native *Eucalyptus* species collected in 13 botanical gardens (Table 1). Leaf inclination angles were measured using a leveled digital camera approach [1]. Images were taken during calm conditions to prevent wind effects on leaves [2]. Depending on the location the images were taken either with a Nikon CoolPix 4500 digital camera (4MP) or a Sony Xperia Z5 Compact phone equipped with 23MP 1/2.3-inch multi-aspect BSI CMOS sensor paired with an F2.0 lens. Leaves were measured in all the azimuth directions as conditions permitted, and along the vertical profile. The data consist of one raw data file (“Pisek_Adamson_2020_DiB.csv”) with 6646 lines (one header line; individual leaf angle measurements) and 10 columns (variables). The data format corresponds to the one used for reporting leaf angle measurements in TRY plant trait database [3]. The column names and definitions of variables are provided in Table 2. The resulting statistical characteristics of leaf inclination angle distributions for each studied species are provided in Table 3 as well as the file “Pisek_Adamson_2020_DiB_processed.csv” in the supplementary material. The statistical characteristics of leaf inclination angle distributions for each studied species were obtained with a R code (“getLIAD.R”), sourced from the original code by [4]. The example input file format (“input_example_LIA.csv”) is also provided.

Table 1. Locations of botanical gardens where the measurements were taken. Lat - Latitude, Lon - Longitude, Date of measurements provided in YYYYMMDD format.

| Botanical garden | Lat | Lon | Date of measurements |
| --- | --- | --- | --- |
| Australian National Botanic Gardens, Canberra, ACT, Australia | -35.276 | 149.108 | 20190222 |
| Blue Mountains Botanic Garden, Mount Tomah NSW, Australia | -33.539 | 150.421 | 20190217 |
| National Arboretum Canberra, Canberra. ACT, Australia | -35.287 | 149.069 | 20190222 |
| Royal Botanic Gardens Victoria - Melbourne Gardens, Melbourne, VIC, Australia | -37.829 | 144.978 | 20130719 |
| Royal Botanic Gardens, Sydney, NSW, Australia | -33.864 | 151.217 | 20190213 |
| Royal Tasmanian Botanical Gardens, Hobart, TAS, Australia | -42.865 | 147.330 | 20130728 |
| The Australian Botanic Garden, Mount Annan, NSW, Australia | -34.071 | 150.766 | 20190224 |
| The Jerusalem Botanical Gardens, Jerusalem, Israel | -31.770 | 35.200 | 20150227 |
| Jardí Botànic de València, València, Spain | -39.477 | -0.386 | 20171107 |
| Royal Botanic Gardens, Kew, the United Kingdom | 51.478 | -0.295 | 20171019 |
| San Francisco Botanical Garden, San Francisco, CA, USA | 37.767 | -122.470 | 20151214 |
| The Huntington Botanical Gardens, Pasadena, CA, USA | 34.128 | -118.116 | 20121209 |
| University of California Botanical Garden at Berkeley, Berkeley, CA, USA | 37.874 | -122.238 | 20131210 |

Table 2. Data description including column names and variable definitions in “Pisek_Adamson_2020_DiB.csv”.

| Column name | Description |
| --- | --- |
| Species | Latin name for the species |
| Latitude | Location latitude (in decimal degrees) |
| Longitude | Location longitude (in decimal degrees) |
| Altitude (m a.s.l.) | Location altitude (in meters above sea level) |
| Sampling Date | (mm/dd/yy) |
| Exposition | botanical garden/alley/natural forest |
| Maturity | mature/seedling |
| Plant Growth Form | mallee, tree, small tree, shrub |
| Comments, Methods | angles measured at whole tree level using leveled digital photo method;  leaf angles reported as differences from a horizontal surface  (i.e. flat horizontal leaf = 0 degrees, vertically oriented leaf = 90 degrees). |
| Reference | Corresponding publication |
| Measurement | in degrees, values 0-90 |

Table 3. Statistical characteristics (i.e., mean, standard deviation) of leaf angle distributions with two parameters μ, ν and classic type of leaf angle distribution of fitted Beta-distributions. PG – plagiophile, U – uniform, S – spherical, Er – erectophile. Table available as “Pisek_Adamson_2020_DiB_processed.csv” in the supplementary material.

| Species name | Measurement location | Count | Mean | S.D. | u | v | Type |
| --- | --- | --- | --- | --- | --- | --- | --- |
| *Eucalyptus albopurpurea* | Jerusalem, IL | 84 | 59.89 | 22.11 | 0.90 | 1.79 | S |
| *Eucalyptus amplifolia* | NBG Canberra, , ACT, AU | 83 | 76.65 | 13.31 | 0.71 | 4.07 | Er |
| *Eucalyptus archeri* | Kew, GB | 90 | 50.78 | 24.60 | 1.00 | 1.29 | U |
| *Eucalyptus baeuerlenii* | NBG Canberra, , ACT, AU | 83 | 69.23 | 21.97 | 0.46 | 1.52 | Er |
| *Eucalyptus balladoniensis* | Pasadena, CA, USA | 83 | 49.68 | 22.57 | 1.31 | 1.62 | U |
| *Eucalyptus benthamii* | Canberra, ACT, AU | 81 | 73.86 | 13.93 | 0.92 | 4.22 | Er |
| *Eucalyptus caesia* | Melbourne, VIC, AU | 100 | 66.58 | 16.67 | 1.20 | 3.41 | Er |
| *Eucalyptus calycogona* | Pasadena, CA, USA | 100 | 40.70 | 24.49 | 1.29 | 1.06 | U |
| *Eucalyptus camaldulensis* | Hobart, TAS, AU | 50 | 69.23 | 18.49 | 0.74 | 2.47 | Er |
| *Eucalyptus camaldulensis var. Acuminata* | Jerusalem, IL | 85 | 72.59 | 12.73 | 1.31 | 5.48 | Er |
| *Eucalyptus chapmaniana* | Kew, GB | 86 | 83.18 | 5.16 | 1.54 | 18.76 | Er |
| *Eucalyptus coccifera* | Kew, GB | 85 | 70.47 | 19.96 | 0.53 | 1.92 | Er |
| *Eucalyptus coolabah* | Canberra, ACT, AU | 87 | 55.01 | 22.71 | 1.06 | 1.67 | S |
| *Eucalyptus copulans* | Mt. Annan, NSW, AU | 80 | 51.74 | 25.81 | 0.84 | 1.13 | U |
| *Eucalyptus crebra* | Mt. Annan, NSW, AU | 83 | 67.80 | 17.95 | 0.91 | 2.77 | Er |
| *Eucalyptus dalrympleana* | Kew, GB | 85 | 79.17 | 10.83 | 0.76 | 5.55 | Er |
| *Eucalyptus decurva* | Berkeley, CA, USA | 66 | 60.04 | 19.80 | 1.20 | 2.40 | S |
| *Eucalyptus delegatensis* | Kew, GB | 78 | 80.87 | 6.31 | 1.78 | 15.76 | Er |
| *Eucalyptus deuaensis* | Sydney, CA, USA | 85 | 48.32 | 27.69 | 0.75 | 0.87 | U |
| *Eucalyptus eremicola* | Jerusalem, IL | 79 | 59.38 | 20.52 | 1.13 | 2.19 | S |
| *Eucalyptus erythrocorys* | Valencia, ES | 80 | 70.84 | 18.94 | 0.59 | 2.19 | Er |
| *Eucalyptus erythronema* | Pasadena, CA, USA | 90 | 43.97 | 24.37 | 1.23 | 1.18 | U |
| *Eucalyptus eximia* | Pasadena, CA, USA | 88 | 75.62 | 13.74 | 0.76 | 4.00 | Er |
| *Eucalyptus ficifolia* | Pasadena, CA, USA | 97 | 57.36 | 19.33 | 1.46 | 2.56 | S |
| *Eucalyptus forrestiana* | Berkeley | 85 | 62.36 | 19.10 | 1.14 | 2.58 | Er |
| *Eucalyptus glaucescens* | Kew, GB | 83 | 70.22 | 18.43 | 0.68 | 2.41 | Er |
| *Eucalyptus gregsoniana* | Kew, GB | 79 | 56.85 | 23.70 | 0.87 | 1.49 | S |
| *Eucalyptus grossa* | Pasadena, CA, USA | 82 | 43.77 | 27.58 | 0.85 | 0.81 | U |
| *Eucalyptus guilfoylei* | Pasadena, CA, USA | 97 | 46.08 | 19.22 | 2.19 | 2.29 | PG |
| *Eucalyptus gunnii* | Kew, GB | 82 | 61.71 | 24.54 | 0.60 | 1.30 | S |
| *Eucalyptus haemastoma* | Mt. Annan, NSW, AU | 85 | 67.29 | 16.51 | 1.16 | 3.44 | Er |
| *Eucalyptus incrassata* | Jerusalem, IL | 88 | 37.95 | 24.28 | 1.36 | 0.99 | U |
| *Eucalyptus intertexta* | Canberra, ACT, AU | 89 | 71.52 | 17.12 | 0.72 | 2.79 | Er |
| *Eucalyptus jacksonii* | San Francisco, CA, USA | 87 | 51.69 | 21.37 | 1.42 | 1.92 | S |
| *Eucalyptus kruseana* | Pasadena, CA, USA | 75 | 57.34 | 22.53 | 0.98 | 1.71 | S |
| *Eucalyptus lacrimans* | Canberra, ACT, AU | 100 | 57.68 | 19.76 | 1.36 | 2.42 | S |
| *Eucalyptus lacrimans* | Pasadena, CA, USA | 75 | 67.78 | 16.64 | 1.10 | 3.34 | Er |
| *Eucalyptus laevopinea* | Mt. Annan, NSW, AU | 82 | 65.64 | 24.86 | 0.43 | 1.16 | S |
| *Eucalyptus langleyi* | Canberra, ACT, AU | 85 | 63.80 | 21.23 | 0.79 | 1.92 | Er |
| *Eucalyptus lansdowneana ssp. Albopurpurea* | Pasadena, CA, USA | 83 | 38.36 | 24.68 | 1.29 | 0.96 | U |
| *Eucalyptus leucoxylon* | Jerusalem, IL | 93 | 53.78 | 25.84 | 0.77 | 1.15 | S |
| *Eucalyptus litorea* | Jerusalem, IL | 91 | 43.00 | 25.78 | 1.07 | 0.98 | U |
| *Eucalyptus macrandra* | Pasadena, CA, USA | 96 | 41.85 | 24.94 | 1.20 | 1.04 | U |
| *Eucalyptus macrocarpa* | Mt. Annan, NSW, AU | 12 | 60.60 | 14.52 | 2.43 | 5.01 | Er |
| *Eucalyptus mannifera* | Canberra, ACT, AU | 84 | 66.25 | 18.48 | 0.95 | 2.66 | Er |
| *Eucalyptus michaeliana* | Canberra, ACT, AU | 81 | 76.59 | 10.45 | 1.25 | 7.16 | Er |
| *Eucalyptus microtheca* | Pasadena, CA, USA | 84 | 77.79 | 10.08 | 1.13 | 7.22 | Er |
| *Eucalyptus nitida* | Kew, GB | 83 | 72.93 | 19.02 | 0.46 | 1.98 | Er |
| *Eucalyptus morrisbyi* | Canberra, ACT, AU | 78 | 58.85 | 24.54 | 0.71 | 1.34 | S |
| *Eucalyptus neglecta* | Kew, GB | 80 | 72.09 | 20.45 | 0.42 | 1.67 | Er |
| *Eucalyptus nicholii* | Canberra, ACT, AU | 88 | 69.57 | 19.72 | 0.60 | 2.05 | Er |
| *Eucalyptus nicholii* | San Francisco, CA, USA | 89 | 72.99 | 19.19 | 0.45 | 1.92 | Er |
| *Eucalyptus oleosa* | Pasadena, CA, USA | 84 | 46.45 | 25.63 | 1.01 | 1.07 | U |
| *Eucalyptus oleosa* | Jerusalem, IL | 100 | 48.19 | 25.18 | 1.01 | 1.17 | U |
| *Eucalyptus parramattensis* | Mt. Annan, NSW, AU | 80 | 74.87 | 17.13 | 0.48 | 2.38 | Er |
| *Eucalyptus parvula* | Kew, GB | 100 | 70.04 | 16.36 | 0.94 | 3.29 | Er |
| *Eucalyptus parvula* | Canberra, ACT, AU | 100 | 52.15 | 22.53 | 1.21 | 1.67 | S |
| *Eucalyptus perriniana* | Kew, GB | 78 | 79.75 | 12.38 | 0.49 | 3.84 | Er |
| *Eucalyptus petiolaris* | Jerusalem, IL | 56 | 38.54 | 22.82 | 1.61 | 1.20 | U |
| *Eucalyptus pleurocarpa* | Mt. Annan, NSW, AU | 76 | 68.87 | 21.47 | 0.51 | 1.65 | Er |
| *Eucalyptus porosa* | Jerusalem, IL | 91 | 68.18 | 19.07 | 0.75 | 2.34 | Er |
| *Eucalyptus pulchella* | Kew, GB | 100 | 67.40 | 17.68 | 0.97 | 2.90 | Er |
| *Eucalyptus pulchella* | Melbourne, VIC, AU | 91 | 49.93 | 21.23 | 1.53 | 1.91 | S |
| *Eucalyptus pulverulenta* | Melbourne, VIC, AU | 58 | 76.94 | 10.82 | 1.10 | 6.49 | Er |
| *Eucalyptus raveretiana* | Mt. Annan, NSW, AU | 78 | 71.18 | 17.35 | 0.72 | 2.73 | Er |
| *Eucalyptus robusta* | Pasadena, CA, USA | 98 | 49.83 | 18.64 | 2.13 | 2.64 | PG |
| *Eucalyptus rodwayi* | Kew, GB | 43 | 63.61 | 21.89 | 0.73 | 1.77 | Er |
| *Eucalyptus scoparia* | Mt. Annan, NSW, AU | 77 | 72.00 | 19.71 | 0.47 | 1.87 | Er |
| *Eucalyptus scoparia* | Canberra, ACT, AU | 77 | 71.13 | 16.10 | 0.88 | 3.30 | Er |
| *Eucalyptus scoparia* | Canberra, ACT, AU | 79 | 78.35 | 10.81 | 0.88 | 5.93 | Er |
| *Eucalyptus shirleyi* | Pasadena, CA, USA | 100 | 48.66 | 21.12 | 1.61 | 1.90 | U |
| *Eucalyptus sideroxylon* | Hobart, TAS, AU | 68 | 64.00 | 18.89 | 1.06 | 2.60 | Er |
| *Eucalyptus stellulata* | Mt. Tobah, NSW, AU | 80 | 77.52 | 12.83 | 0.68 | 4.20 | Er |
| *Eucalyptus stoatei* | Pasadena, CA, USA | 86 | 42.43 | 25.24 | 1.15 | 1.02 | U |
| *Eucalyptus stricta* | Canberra, ACT, AU | 86 | 40.42 | 24.04 | 1.36 | 1.11 | U |
| *Eucalyptus subcrenulata* | Kew, GB | 80 | 56.85 | 25.14 | 0.73 | 1.25 | S |
| *Eucalyptus tereticornis* | Mt. Annan, NSW, AU | 120 | 70.62 | 17.29 | 0.77 | 2.81 | Er |
| *Eucalyptus tricarpa* | Canberra, ACT, AU | 80 | 64.09 | 22.36 | 0.67 | 1.65 | Er |
| *Eucalyptus urnigera* | Kew, GB | 80 | 64.85 | 22.35 | 0.63 | 1.63 | Er |
| *Eucalyptus viridis* | Jerusalem, IL | 75 | 42.19 | 25.85 | 1.07 | 0.95 | U |

**Experimental Design, Materials and Methods**

*Leaf inclination measurements and data processing*

The method proposed by [1] consists of acquiring leveled images of the canopy with a digital camera. A minimum of 75 leaf inclination angle measurements shall permit a statistically representative sample to characterize the leaf inclination angle distribution [5]. It shall be noted that the method is suited to broadleaf plant species [6]. The identification of the leaf plane, from which the leaf normal is measured, is required for the measurement of leaf inclination angle (Fig. 1). For this reason, the leaves oriented approximately perpendicular to the viewing direction of the camera (i.e., the leaves shown as a line in the image; Fig. 1) were selected for measurement of leaf angles. The leaf angles were measured using the ‘angle measurement tool’ of the freeware program ‘ImageJ’ (<http://rsbweb.nih.gov/ij/)>. Although some level of uncertainty might be still present in individual leaf measurements due to user’s subjectivity, the method was found quite robust in providing the same distributions of De Wit [7] irrespective of the user and their previous experience with measuring leaf inclination angles [8].

*Estimation and assignment of beta distribution type*

The measured leaf inclination angles were used to estimate the leaf inclination angle distribution for each species. A two-parameter Beta distribution [9] was previously identified as the most appropriate distribution to represent the probability density of $\theta$*_L_* [10]:

$f\left( t \right)=\frac{1}{B\left( \mu,\nu\right)}{(1-t)}^{\mu-1}t^{\nu-1}$ (1)

where *t*=2 *θ_L_/π* and θ*_L_* is expressed in radians. The Beta distribution *B(μ,ν)* is defined as:

$B\left( \mu,\nu\right)=\int_{0}^{1} {(1-x)}^{\mu-1}x^{\nu-1}dx=\frac{\Gamma(\mu)\Gamma(\nu)}{\Gamma(\mu+\nu)}$ (2)

where Γ is the Gamma function and *μ* and *ν* are two parameters of the Beta distribution, which are calculated as:

$\mu=(1-\bar{t})\left( \frac{\sigma_{0}^{2}}{\sigma_{t}^{2}}-1 \right)$ (3)

$\nu=\bar{t}\left( \frac{\sigma_{0}^{2}}{\sigma_{t}^{2}}-1 \right)$ (4)

where $\sigma_{0}^{2}$ is the maximum standard deviation with an expected mean $\bar{t}$; $\sigma_{t}^{2}$ is the variance of *t* [10].

Leaf inclination angle distributions can be described with six common functions [7]: planophile, plagiophile, uniform, spherical, erectophile and extremophile. Horizontally oriented leaves are dominant in planophile canopies; plagiophile canopies are dominated by inclined leaves; uniform canopies possess about an equal proportion of leaf inclination angles for any angle; in spherical canopies, the relative frequency of leaf inclination angle is the same as for a sphere; erectophile canopies are dominated by vertically oriented leaves; extremophile distribution is a rather theoretical case, which would be characterized by both horizontally and vertically oriented leaves. All measured leaf inclination angle distributions were additionally classified by assigning them to the closest classical distribution type, since the classical distributions are widely used and easier to interpret than the Beta distribution parameters. Deviation of each leaf inclination angle distribution from the distributions suggested by de Wit $f_{de Wit}\left( \theta_{L} \right)$was quantified with a modified version of the inclination index provided by [11]:

$\chi_{L=} \int_{0}^{\pi/2} \left| f\left( \theta_{L} \right)-f_{de Wit}\left( \theta_{L} \right) \right|d\theta_{L}$ (5)

RStudio Version 1.0.153 has been used for all the data processing described above.

**Acknowledgments**

The data were collected as a part of projects from Estonian Research Council Grants PUT 232, PUT1355, Mobilitas Pluss MOBERC11, P7-Marie Curie Actions program, and Estonian Science Foundation grant no. ERMOS32. We thank Dr. Heleri Ramler for proof-reading revised version of the manuscript. We thank two reviewers for their constructive comments and provided tools.

**Supplementary material**

Supplementary material associated with this article can be found in the online version.

**Declaration of Competing Interest**

The authors declare that they have no known competing financial interests or personal relationships which have or could be perceived to have influenced the work reported in this article.

**References**

[1] Y. Ryu, O. Sonnentag, T. Nilson, R. Vargas, H. Kobayashi, R. Wenk, D.D. Baldocchi, 2010. How to quantify tree leaf area index in a heterogeneous savanna ecosystem: a multi-instrument and multi-model approach. Agric. For. Meteorol. 150, 63–76.

[2] L. Tadrist, M. Saudreau, E. de Langre, 2014. Wind and gravity mechanical effects on leaf inclination angles. J. Theor. Biol. 341, 9–16.

[3] J. Kattge, G. Boenisch, S. Diaz, S. Lavorel, I.C. Prentice, P. Leadley; S. Tautenhahn, G.D.A. Werner et al., 2020. TRY plant trait database - enhanced coverage and open access. Global Change Biology, 26, 119−188.10.1111/gcb.14904.

[4] F. Chianucci, J. Pisek, K. Raabe, L. Marchino, C. Ferrara, P. Corona, 2018. A dataset of leaf inclination angles for temperate and boreal broadleaf woody species. Ann. For. Sci. 75(2): 50. doi:10.1007/s13595-018-0730-x.

[5] J. Pisek, O. Sonnentag, A.D. Richardson, M. Mõttus, 2013. Is the spherical leaf inclination angle distribution a valid assumption for temperate and boreal broadleaf tree species? Agric. For. Meteorol. 169, 186–194.

[6] F. Chianucci, 2020. An overview of in situ digital canopy photography in forestry. Canadian Journal of Forest Research, 50, 227-242.

[7] C.T. de Wit, 1965. Photosynthesis of leaf canopies. In: Agricultural Research Report No. 663. Wageningen, http://library.wur.nl/WebQuery/wurpubs/413358

[8] K. Raabe, J. Pisek, O. Sonnentag, K. Annuk, 2015. Variations of leaf inclination angle distribution with height over the growing season and light exposure for eight broadleaf tree species. Agric. For. Meteorol. 214–215: 2–11.

[9] N.S. Goel, D.E. Strebel, 1984. Simple beta distribution representation of leaf orientation in vegetation canopies. Agron. J. 76, 800–802.

[10] W.M. Wang, Z.I. Li, H.B. Su, 2007. Comparison of leaf angle distribution functions: effects on extinction coefficient and fraction of sunlit foliage. Agric. For. Meteorol. 143, 106–122.

[11] J. Ross, 1975. Radiative transfer in plant communities. In: Monteith, J.L. (Ed.), Vegetation and the Atmosphere, vol. 1. Academic Press, London, UK, pp. 13–55.

**Figure**


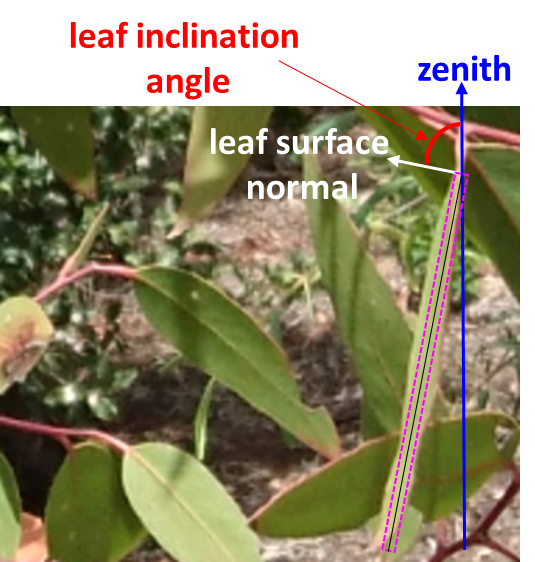


Figure 1. A schematic diagram of the protocol used to measure leaf inclination angle from leveled digital photography. The leaf plane is indicated by the line in a purple box.
